# Supplementary material for: Transcription Factor Condensates Mediate Clustering of MET Regulon and Enhancement in Gene Expression
Source: bioRxiv. 2024 Jul 12:2024.02.06.579062. Originally published 2024 Feb 8. Preprint. [Version 2] doi: 10.1101/2024.02.06.579062 (PMC10871269; doi:10.1101/2024.02.06.579062)
Supplement: Supplement 1 [file media-1.pdf]

Supplemental Figure S1

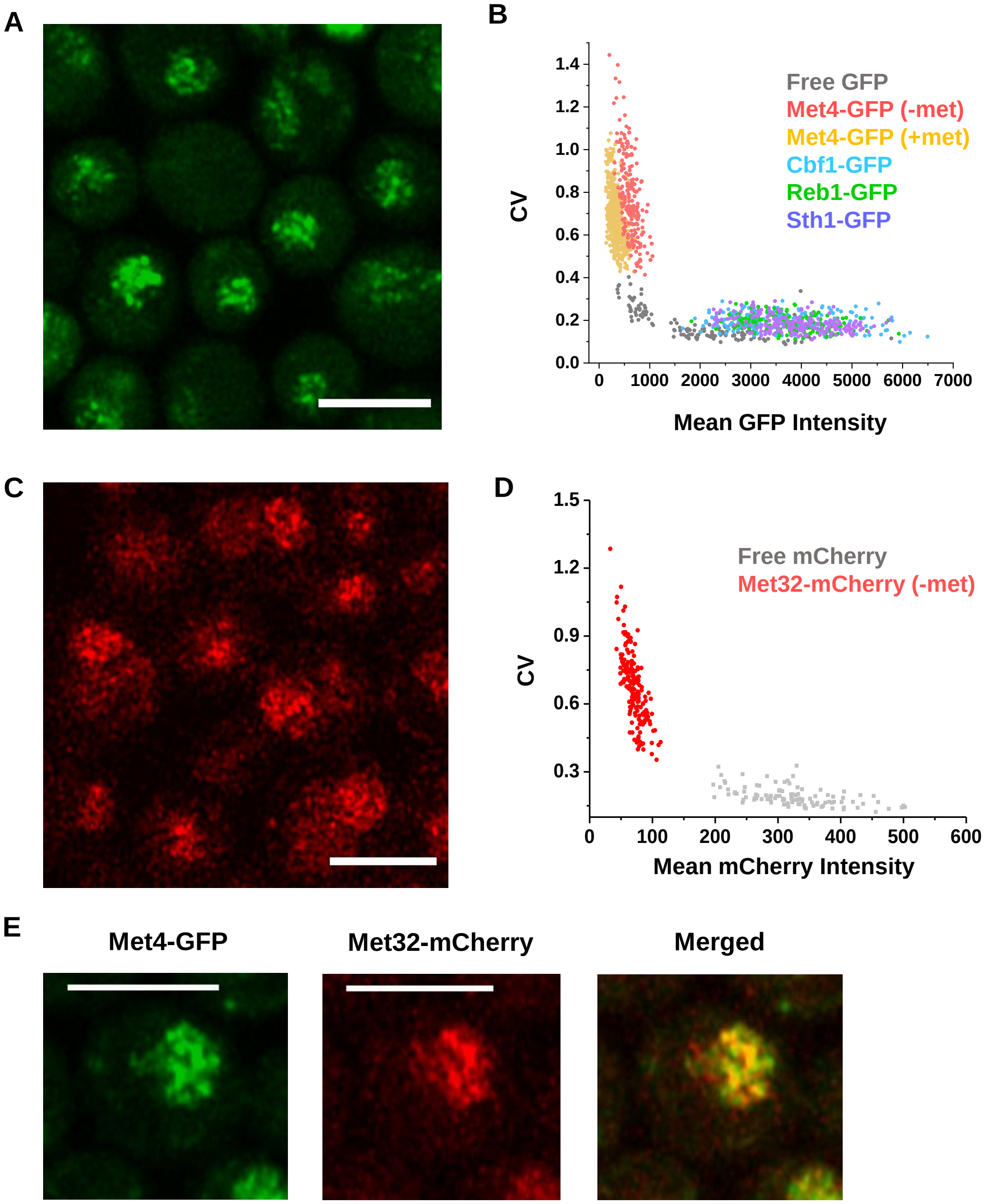

Figure S1. A) Additional images of Met4-GFP in live yeast cells under –met condition. Scale bars represent 4 µm. Same for below. **B)** CV of pixel intensities vs average intensity in cells expressing various GFP fusions as in Fig 1A. Each dot represents a single nucleus. CV decreases with increasing GFP concentration, and different concentrations of different factors are taken into account by the Fano number. **C)** Additional images of Met32-mCherry in live yeast cells under –met condition. **D)** Same as in B for free mCherry and Met32-mCherry. **E)** Additional images of Met4-GFP and Met32-mCherry co-expressed in live yeast cells under –met condition.

Supplemental Figure S2

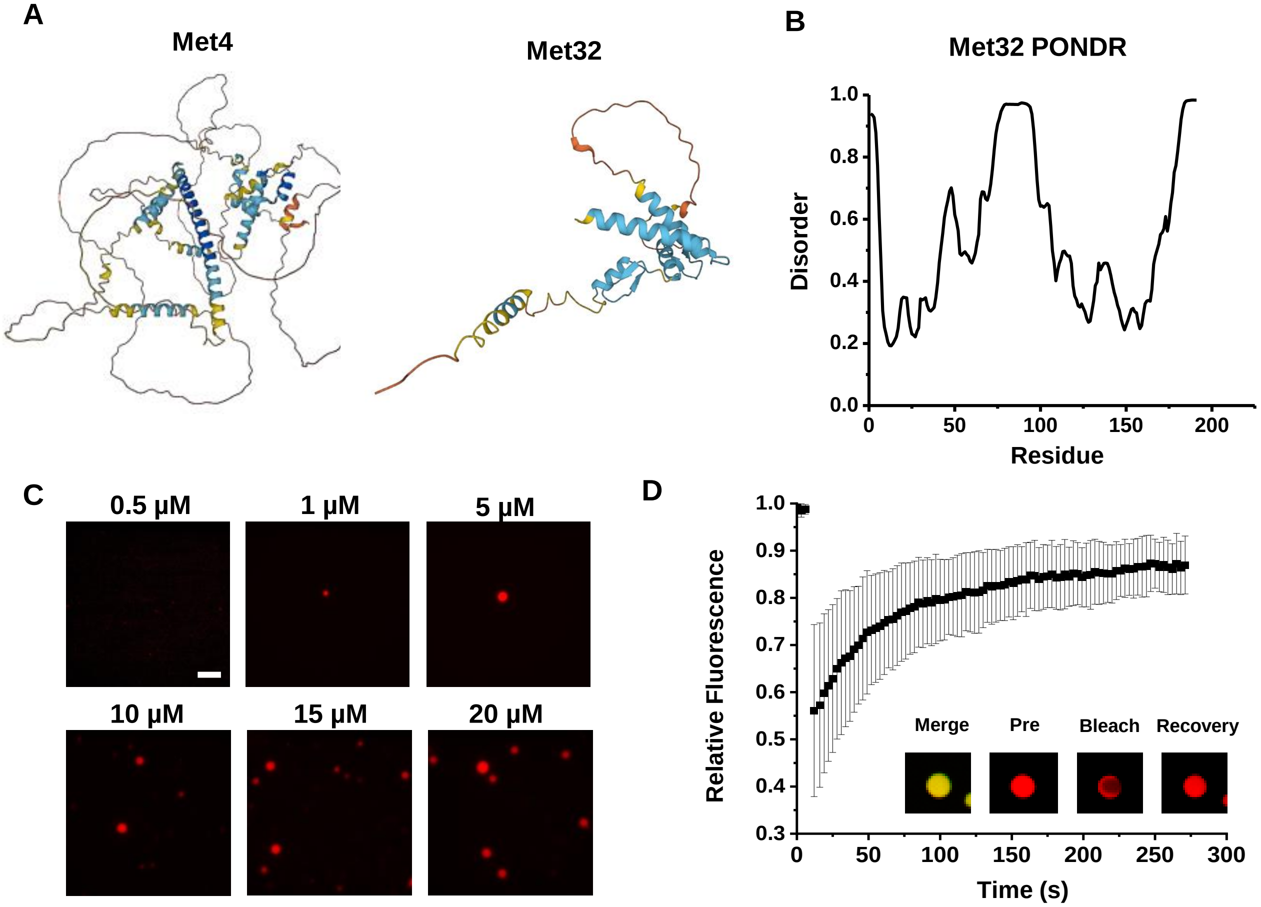

Figure S2. A) Predicted protein structures of Met4 (Alphafold: AF-A0A1L4AA63-F1) and Met32 (AF-A0A0L8VTL7-F1). **B)** PONDR protein disorder plot of Met32 (same analysis for Met4 is shown in Fig. 7). **C)** Met32-mCherry condensate formation at various concentrations. Purified Met32-mCherry proteins were titrated in 20mM HEPES pH 7.5, 150 mM Nacl. Scale bar represents 10 µm. **D)** FRAP curve for Met4 / Met32 co-localized droplets. 5 µM Met4-MBP and 20 µM Met32-mCherry were mixed. Intensity data collected every 3 seconds for 270 seconds.

Supplemental Figure S3

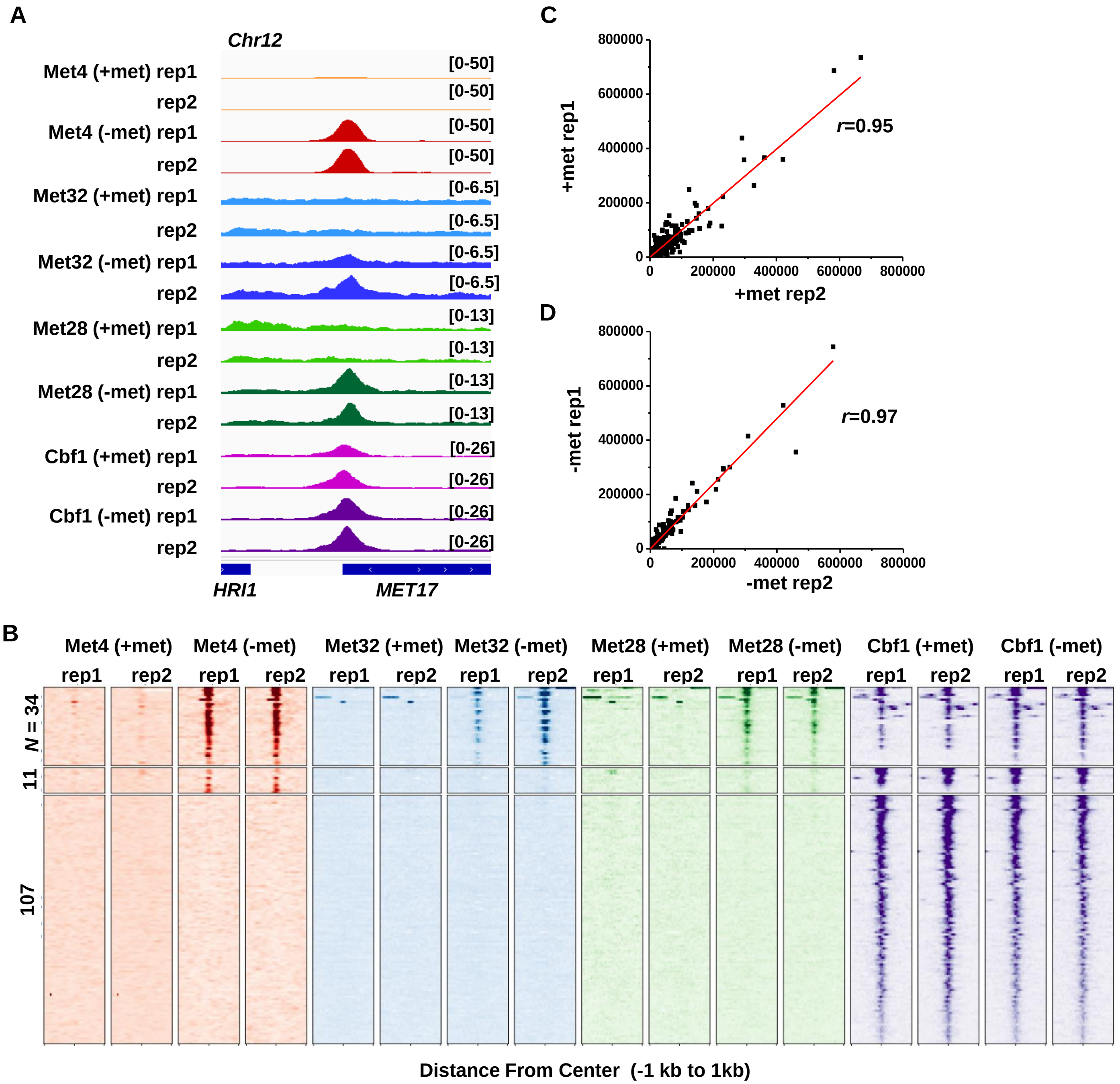

Figure S3. A) Additional examples of ChIP-seq data on Met TFs (Met4, Met32, Met28 and Cbf1). For each factor, two biological replicates in ±met conditions are shown. In this case, the Met TF co-binding peak is in the *MET17* promoter. **B)** Same as in Figure 3C but showing two replicates of the ChIP-seq data for the four factors in ± met conditions. **C & D)** Comparison of read counts from two biological replicates of RNA-seq data in +/- met conditions. Pearson's correlations (*r*) between the two biological replicates are shown.

Supplemental Figure S4

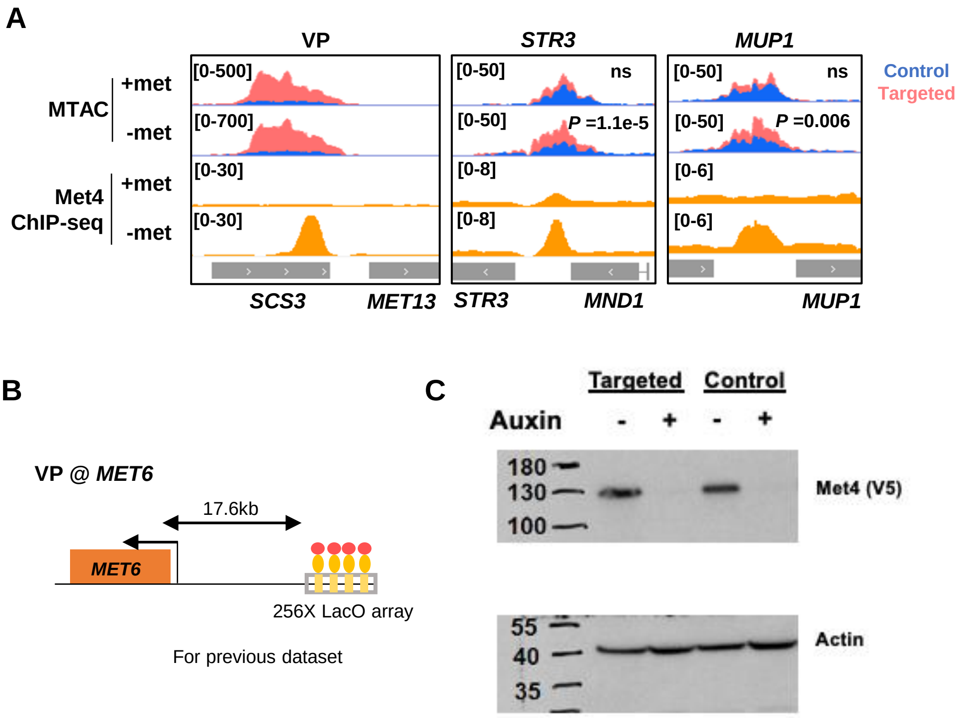

Figure S4. A) MTAC and Met4 ChIP-seq data at the *MET13* locus (VP), as well as at *STR3* and *MUP1* as interacting regions of the VP. MTAC signal and ChIP enrichment are both shown in ± met conditions. *P,* FDR-adjusted *P* value, Wald test by DESeq2. ns, non-significant. **B)** VP design near the *MET6* locus used for Figure 4D-F. **C)** Western blot of V5-AID-tagged Met4 ± Auxin(IAA) in MTAC targeted and control strains. Actin is probed as input loading control.

Supplemental Figure S5

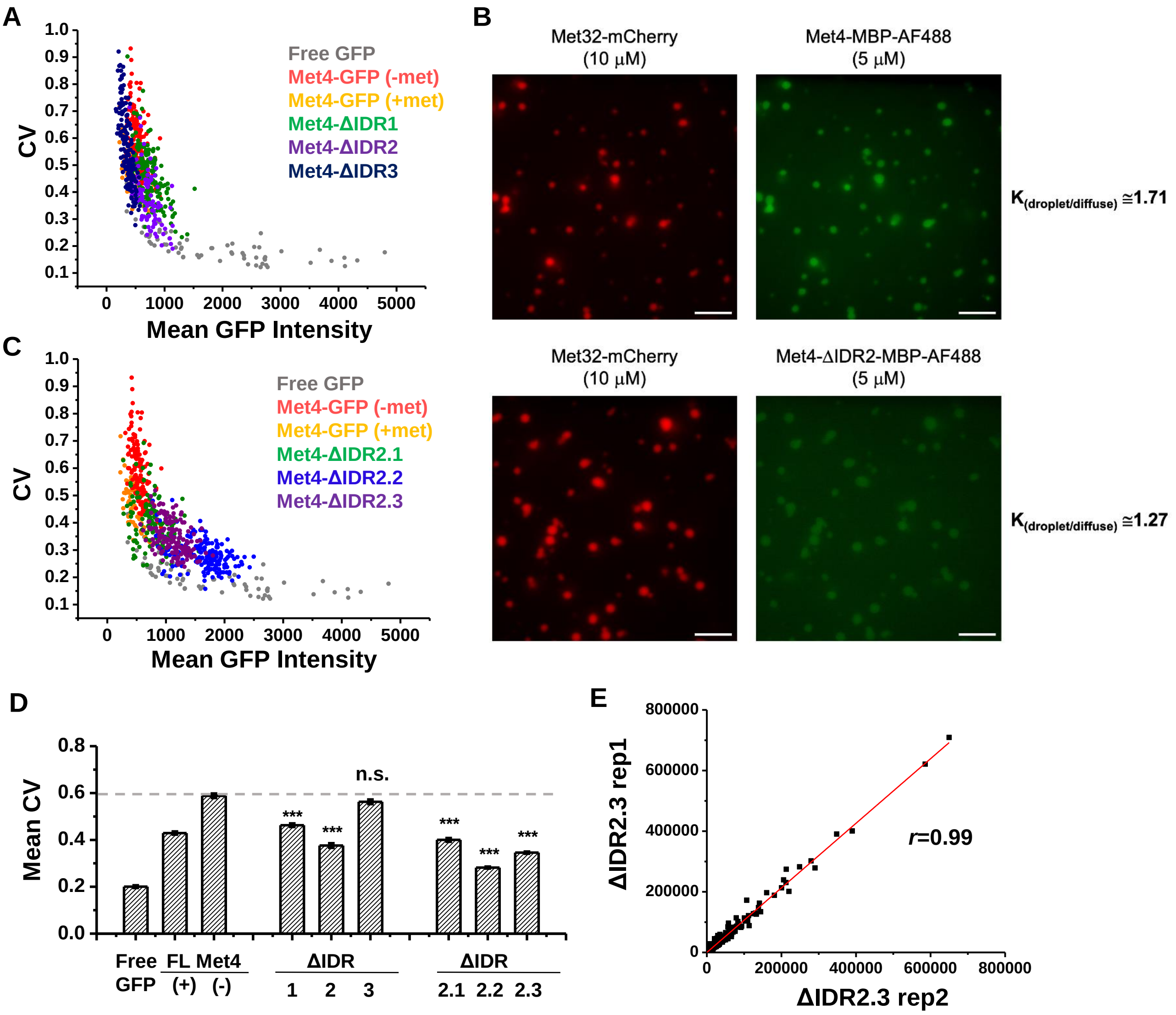

Figure S5. A) CV of pixel intensities vs average intensity in cells expressing free GFP or GFP fused with full length Met4 (in ± met), ΔIDR1 (aa1-69), ΔIDR2 (aa117-359), and ΔIDR3 (aa397-651). Individual dots represent single nuclei. IDR strains were grown in SCD-met. **B)** Partitioning of full length Met4 and Met4-ΔIDR2 into the Met32 condensates. The ratios of the green fluorescent intensity inside vs outside the Met32 droplet are shown on the right. **C)** Same as in A except with Met4 ΔIDR2.1 (aa117-135), ΔIDR2.2 (aa136-270), and ΔIDR2.3 (aa271-359). IDR strains were grown in SCD-met. **D)** The mean CV values of nuclear pixel intensities of different GFP fusions in panel A and B. P-values were calculated against the Free GFP. **E)** Comparison of read counts from two biological replicates of RNA-seq data of the ΔIDR2.3 strain in -met. Pearson's correlations (*r*) between the two biological replicates are shown.
